# Adoptive Immunotherapy beyond CAR T-Cells

**DOI:** 10.3390/cancers13040743

**Published:** 2021-02-11

**Authors:** Aleksei Titov, Ekaterina Zmievskaya, Irina Ganeeva, Aygul Valiullina, Alexey Petukhov, Aygul Rakhmatullina, Regina Miftakhova, Michael Fainshtein, Albert Rizvanov, Emil Bulatov

**Affiliations:** 1 Institute of Fundamental Medicine and Biology, Kazan Federal University, 420008 Kazan, Russia; titov.a@blood.ru (A.T.); EAZmievskaya@kpfu.ru (E.Z.); IAGaneeva@kpfu.ru (I.G.); AjgHValiullina@kpfu.ru (A.V.); AjgulRRahmatullina@kpfu.ru (A.R.); ReRMiftahova@kpfu.ru (R.M.); Albert.Rizvanov@kpfu.ru (A.R.); 2 Laboratory of Transplantation Immunology, National Hematology Research Centre, 125167 Moscow, Russia; 3 Institute of Hematology, Almazov National Medical Research Center, 197341 Saint Petersburg, Russia; petukhov_av@almazovcentre.ru; 4 Mircod Biotech Inc., Boca Raton, FL 33432, USA; michael@mircod.com; 5 Shemyakin-Ovchinnikov Institute of Bioorganic Chemistry, Russian Academy of Sciences, 117997 Moscow, Russia

**Keywords:** chimeric antigen receptor, CAR T-cell, CAR NK-cell, transgeneic TCR, TIL, neoantigen, neoepitope, peptide

## Abstract

**Simple Summary:**

The aging of the world population leads to a constant increase of cancer-related morbidity and mortality. Treatment of late-stage tumors has become a significant burden on the healthcare system globally. Adoptive cell immunotherapy is supposed to prolong life with cancer and ideally cure cancer after a single infusion of the cell product. Arguably, the most impressive clinical therapy in this field is based on chimeric antigen receptor (CAR) T-cells capable of curing up to 25–50% of previously incurable patients with B-cell malignancies. Diverse cell therapies are already efficiently used in clinics for cancer treatment (such as tumor infiltrating lymphocytes, transgenic αβ T-cells) and several novel promising cell therapies are in development (such as CAR M-cells, transgenic γδ T-cells, CAR NK-cells). Here, we summarize the recent literature data with the focus on T-cell receptor-based therapies and overview the most advanced systems for manufacturing of clinical grade cell products.

**Abstract:**

Adoptive cell immunotherapy (ACT) is a vibrant field of cancer treatment that began progressive development in the 1980s. One of the most prominent and promising examples is chimeric antigen receptor (CAR) T-cell immunotherapy for the treatment of B-cell hematologic malignancies. Despite success in the treatment of B-cell lymphomas and leukemia, CAR T-cell therapy remains mostly ineffective for solid tumors. This is due to several reasons, such as the heterogeneity of the cellular composition in solid tumors, the need for directed migration and penetration of CAR T-cells against the pressure gradient in the tumor stroma, and the immunosuppressive microenvironment. To substantially improve the clinical efficacy of ACT against solid tumors, researchers might need to look closer into recent developments in the other branches of adoptive immunotherapy, both traditional and innovative. In this review, we describe the variety of adoptive cell therapies beyond CAR T-cell technology, i.e., exploitation of alternative cell sources with a high therapeutic potential against solid tumors (e.g., CAR M-cells) or aiming to be universal allogeneic (e.g., CAR NK-cells, γδ T-cells), tumor-infiltrating lymphocytes (TILs), and transgenic T-cell receptor (TCR) T-cell immunotherapies. In addition, we discuss the strategies for selection and validation of neoantigens to achieve efficiency and safety. We provide an overview of non-conventional TCRs and CARs, and address the problem of mispairing between the cognate and transgenic TCRs. Finally, we summarize existing and emerging approaches for manufacturing of the therapeutic cell products in traditional, semi-automated and fully automated Point-of-Care (PoC) systems.

## 1. Introduction

Adoptive immunotherapy is a highly potent option for the treatment of tumors resistant to the current standards of care. Chimeric antigen receptor (CAR) T-cell therapy—one of the brightest success stories in this field—has revolutionized the treatment of resistant hematological malignancies and quickly became a new standard of treatment for relapsed/refractory disease. Nevertheless, the success of CAR T-cell therapy for the treatment of solid tumors is still relatively modest. The caveats may lie in the difficulty of T-cell trafficking into solid tumor tissues due to the stromal barriers, tumor microenvironment, and certain tumor mutations resulting in activation of T-cell exclusion and exhaustion mechanisms. The second important challenge for successful application of CAR T-cell immunotherapy against solid tumors is a limited number of surface tumor-specific targets. Well-studied T-cell receptor and Natural Killer (NK) cell receptor-based technologies are highly promising in expanding the range of potential tumor-specific targets. In addition, novel approaches addressing the trafficking issues were recently proposed (e.g., macrophage-derived CAR M-cells). Numerous institutions and companies around the globe are working on resolving the immunotherapy accessibility issues by developing universal allogeneic cell technologies through exploitation of alternative cell sources or downregulation of cognate T-cell receptors (TCRs). The same problem is being addressed through development of automated Point-of-Care (PoC) systems for manufacturing of cell products that allow easier Good Manufacturing Practice (GMP) and biosafety compliance in comparison with the traditional bioreactors. In this review, we summarize the variety of cell therapy approaches and strategies beyond CAR T-cells and provide a critical assessment of their applicability, as well as the advantages and issues associated with them.

## 2. The Importance of Cell Source for Production of Conventional CAR T-Cells

The manufacturing of currently approved CAR T-cell therapies results in highly heterogeneous cell products. Kymriah (by Novartis) and Yescarta (by Gilead) are produced from bulk mononuclear fraction and do not undergo any T-cell selection step. In July 2020, Tecartus (brexucabtagene autoleucel) by Gilead became the third clinically approved CAR T-cell therapy and is used for the treatment of relapsed mantle cell lymphoma. The primary difference between Yescarta and Tecartus consists in the T-cell selection step that allows depletion of circulating tumor B-cells to prevent their viral transduction and, therefore, potential tumor resistance [1]. Importantly, production of neither of the approved CAR T-cell therapies involves any further T-cell subtype selection (e.g., CD4^+^, CD8^+^, or specific T-cell memory subsets) or depletion (Treg). Thus, the precise dosage of fully functional and potent cells within the product gets specified only directly before the infusion.

The preference of several T-cell subpopulations for production of potent CAR T-cells has been previously discussed in the literature. Several studies report a variety of T-cell subpopulations, such as central memory T-cells (TCMs) [2], stem or stem-like memory T-cells (TSCMs) [3,4,5], CD27^+^CD45RO^−^CD8^+^ cells [4], IL17A-producing polyfunctional CD4^+^ T-cells [6], or defined CD4^+^:CD8^+^ (e.g., 1:1) composition [7,8] that are associated with enhanced in vitro or clinical efficacy. The unequal anti-tumor potential of certain CAR T-cell clones can be illustrated by a significant reduction of CAR T-cell clonal diversity in peripheral blood over time and eventual dominance of a small number of clones [9]. Moreover, in one particular case, the tumor was completely eliminated by a single T-cell clone that underwent tremendous expansion due to occasional disruption of the *TET2* gene after lentiviral vector integration [10] without any signs of the insertional oncogenesis after 4.2 years. This report demonstrated that a single pre-selected or genetically enhanced CAR T-cell can potentially proliferate and provide a robust anti-tumor response. Therefore, safety and efficacy of the therapy to some extent relies on the careful choice of T-cell subsets for introduction of CAR and other genetic modifications [11]. Moreover, the defined CD4^+^:CD8^+^ (e.g., 1:1) composition provides additional capabilities by introduction of different CAR designs to each of the cell subtypes. The functional utility of such a complex design was proved by Guedan et al., who showed that ICOS-costimulated CD4^+^ CAR T-cells significantly improved the persistence of CD8^+^ CAR T-cells with other costimulatory domains in mice [12]. Such observations may have a significant impact on the prospective designs of clinical grade CAR T-cell products.

**Figure 1:**
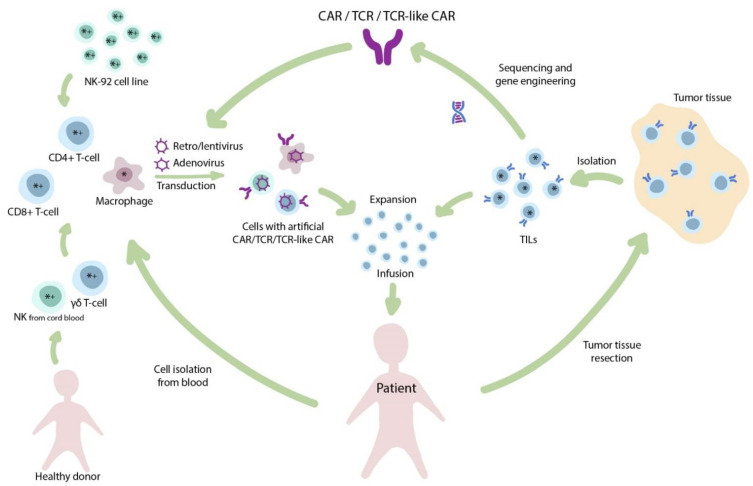
The diverse nature of adoptive immunotherapy. Infusion of tumor infiltrating lymphocytes (TILs) is one of the oldest clinical approaches in T-cell-based immunotherapy. The isolated TILs are expanded ex vivo and infused back to the patient. In addition, TILs and other T-cells may be used for isolation of T-cell receptors (TCRs) for further genetic engineering and generation of transgenic TCR therapies. Alternatively, to widespread CAR T-cells, other cell populations (e.g., NK-cells, macrophages) may be transduced to produce CAR NK-cells and CAR M-cells, respectively. Cellular therapies that are primarily applied for the treatment of solid tumors (*) or hematological malignancies (+).

Within the context of solid tumors and the associated immunosuppressive microenvironment, T-cells demonstrate reduced trafficking as well as enhanced inhibition and exhaustion [13]. Given that T-cells are not the only cell subtype suitable for CAR introduction, other cell populations related to the innate immunity should be carefully considered (Figure 1).

## 3. CAR Cells, but Not CAR αβ T-Cells

### 3.1. γδ. T-Cells

The specific γδ T-cell subset attracts a growing interest in the scientific community since these cells generally recognize non-protein ligands, stress-ligands, and metabolites represented by non-conventional MHC such as CD1c or CD1d [14], as well as butyrophilin-2A1 (BTN2A1) [15] and other molecules. The British TC Biopharm and the Dutch Gadeta exploit the opportunities of this cell type for TCR and CAR-based therapies. The major advantage of these cells is stipulated by the decreased risk of autoimmune complications due to their outstanding cognate specificity (HLA-independent recognition of a narrow range of highly conservative antigens).

### 3.2. NK-Cells

NK-cells in contrast to T-cells recognize the presence of self-MHC class I molecules and detect stress-induced ligands on the tumor cells. However, NK-cells represent a promising cell source for CAR-based therapy because similarly to T-cells, they are capable of perforin/granzyme-dependent cytotoxicity that is regulated by activation and inhibition of surface NK-cell receptors. Interestingly, the cytokine-activated autologous peripheral blood NK-cells may possess substantial therapeutic potential even in the absence of any genetic modification (e.g., introduction of CAR), as was supported by demonstration of measurable clinical benefit in patients with relapsed glioblastoma [16,17]. The clinical benefit from application of genetically unmodified allogeneic NK-cells appears to be more prominent due to a mismatch in repertoire of killer cell immunoglobulin-like receptors (KIR) [18]. The therapeutic depletion of graft αβ T-cells prior to allogeneic hematopoietic stem cell transplantation (HSCT) is supported by the assumption that the primary graft-versus-leukemia effect originates from NK-cells and γδ T-cells rather than αβ T-cells. The follow-up of αβ and CD19^+^ depleted haploidentical HSCT demonstrated lower probability of chronic graft-versus-host disease (GvHD) with comparable leukemia-free survival in a multicenter retrospective trial in Italy with 343 participants [19] and some other smaller trials [20,21]. Moreover, some KIR allelic variants, such as KIR2DS4∗ 00,101 or KIR2DS2, are found to be protective in glioblastoma, while being associated with prolonged overall survival (OS) or reduced risk of glioblastoma in healthy donors [22]. Similarly, to γδ T-cells, the advantage of NK-cells as a cell source for universal allogeneic therapy is their MHC-independence and no need for knockout of endogenous TCR. On the other hand, the proliferative capacity of NK-cells is lower than that of T-cells, therefore making isolation and expansion of NK-cells to sufficient numbers a challenging task.

In a clinical setting, the increased attention to allogeneic NK-cells, e.g., based on the NK-92 line initially derived from a lymphoma patient, is explained by a limited proliferative capacity of primary donor-derived NK-cells. Obviously, such allogeneic cells require prior irradiation to prevent their inoculation leading to formation of secondary tumors. Preclinical [23] trials demonstrated the increased in vitro and in vivo activity of HER2-specific CAR-NK-92 cells in comparison to the parental NK-92. Further, a number of clinical trials is ongoing and a case report of successful treatment of relapsed glioblastoma patients with surgery and HER2-specific CAR-NK-92 cells was presented by Dr. W. Wels to illustrate phase I CAR2BRAIN (NCT03383978) clinical trial [24]. The therapeutic application of CD33-CAR-NK-92 cells in Phase I trial with dose up to 5 × 10^9^ cells was shown to be safe although the clinical benefit was only moderate (significant but transient reduction in blast count) suggesting its most appropriate application as a “bridge” to allogeneic HSCT [25].

Exploiting cord blood allogeneic donor-derived NK-cells allowed Liu et al. at MD Anderson Cancer Center to demonstrate the preliminary efficacy of CAR NK-cells to be almost equivalent to CAR T-cells, at least for B-cell malignancies and in a short period of time [26]. CAR-transduced cord blood NK-cells are designed to produce IL-15 for self-stimulation and, in contrast to NK-92, lack tumorigenic potential that allows omission of the irradiation step during preparation of the therapeutic cell product. The results of the Phase I/II trial revealed 8 out of 11 responses and 7 complete remissions, while some patients demonstrated persistence of the minimal residual disease (MRD)—a small number of tumor cells in the blood, as confirmed by flow cytometry. The complexity of clinical implementation might explain the fact that to date, there are only six ongoing clinical trials with NK-92 cells and only two trials with cord blood NK cells.

### 3.3. CAR M-Cells

Low penetration capability of CAR T-cells into the solid tumor tissues is well known and was extensively discussed in the literature [13,27,28,29]. Adusumilli et al. report observations from a preclinical model demonstrating that intrapleurally administration of CAR T-cells specific to mesothelin required up to 30-fold fewer cells to induce long-term complete remission compared to systemically infused CAR T-cells [30]. This issue of CAR T-cell therapy was addressed in an immunotherapy approach developed specifically for the treatment of solid tumors whereby an adenoviral first-generation CAR vector is transduced in human macrophages resulting in CAR M-cells [31]. These macrophage-derived cells were capable of efficient trafficking to the tumor site, target-specific phagocytosis and reprogramming of the tumor microenvironment (TME) while retaining M1 self-polarization. Importantly, it was found that CAR M-cells are able to induce epitope spreading, the expansion mechanism of tumor-specific T-cells that functions through enhanced processing and presentation of tumor epitopes as a result of phagocytosis by CAR M-cells. Overall, CAR-transduced macrophages significantly improved the survival rates of immunodeficient NOD/SCID mice. Moreover, they have a potential for even stronger impact on the survival of immunocompetent mice with functional T-cells due to the epitope spreading effect [32].

With regards to potential clinical application, macrophages are known to be a hardly susceptible for lentiviral transduction due to the low proliferations rates [33,34]. In ovine models, the efficient transduction was achieved only at very high multiplicity of infection (MOI) values of 10–60 and with the use of polycations like hexadimethrine bromide (polybrene) or protamine sulfate [35]. For CAR M-cells, Klichinsky et al. demonstrated high transduction efficiency using adenoviral vector (serotype 5) [31]. In principle, adenoviral vectors appear to be safe and are used in clinic for oncolytic viral therapy [36] and as vaccines, including anti-SARS CoV-2 [37,38]. However, inability of adenovirus to integrate the cell genome might result in low persistence of CAR expression in adenovirus-transduced macrophages. In addition to that, such critical points as the number of cells per infusion and the potential toxicity remain to be more thoroughly investigated in future clinical trials.

To summarize, further development of the advanced ACT approaches mentioned above is particularly important in the context of two primary issues associated with CAR T-cell therapy: (i) limited affordability for patients due to the high costs of personalized production and the lack of universal allogeneic clinical-stage therapies; (ii) low efficacy against solid tumors (e.g., glioblastoma or pancreatic cancer) that can be potentially resolved by using CAR M-cells associated with enhanced tumor penetration and positive impact on endogenous T-cell response.

## 4. T-Cells, but Not CAR T-Cells. TCR-Based Therapies

### 4.1. The Basis of the TCR Machinery

**Figure 2:**
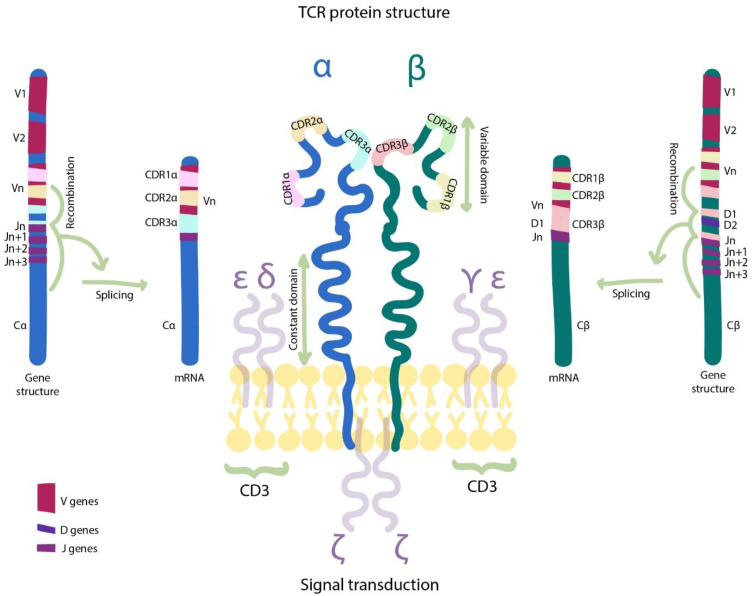
The structure of TCR α and β chains. The TCR constant domains are responsible for the correct complex assembly and binding to CD3 chains, whereas the variable domains are involved in recognition of the peptide–MHC complex. Complementary Determining Regions 3 of α (CDR3α) and β (CDR3β) chains are assembled during somatic mutagenesis and V(D)J recombination and are the most important for antigen recognition. The other CDRs (V-gene specific) are germline encoded and contribute to peptide–MHC recognition.

The functional TCR complex (Figure 2) is assembled from α and β chains each consisting of constant and variable domains. The constant domain is involved in correct assembling and functioning of the TCR complex together with CD3 chains. Recognition of the cognate MHC-peptide complex is fully dependent on the variable TCR chains that are formed during the so-called V(D)J recombination at an early stage of T-cell maturation. Each chain includes derivatives of the V gene, the J gene, and often the D gene between V and J genes. This recombination creates an individual set of V, J, and sometimes D genes in each of the TCR chains. The number of V and J genes is relatively small and V genes distinguish themselves mainly through complementarity-determining regions (CDRs), specifically CDR1 and CDR2 loci. The most hypervariable CDR3 locus is formed by all three V, (D) and J fragments as well as by additional random modification at the gene termini (P/N additions) [39]. The CDR3 fragments of α (CDR3α) and β (CDR3β) TCR chains determine the epitope-specificity while CDR1 and CDR2 are responsible for interaction with HLA. Still the role of each chain in functioning of the whole TCR complex is substantially more complex. It was previously demonstrated that for TCRs recognizing distinct peptides within the same HLA, swapping of CDR3 regions was not sufficient to recapitulate the CDR3-cognate peptide reactivity, even when both TCRs included identical Vα chains [40]. CDR1 and CDR2 fragments can directly interact with the antigen peptide or rearrange MHC conformation for an appropriate pMCH-CDR3 interaction [41], whereas CDR3α region can directly affect the MHC-restricted antigen recognition [42].

During the thymic selection TCR repertoire narrows substantially due to elimination of T-cells bearing TCRs with very strong binding to the self-peptide/MHC complex (negative selection that excludes potentially auto-reactive T-cells) and those with very weak binding (positive selection that excludes non-functional T-cells incapable of self-peptide/MHC binding). Importantly, CD8 TCR co-receptor of the CD8^+^ T-cells binds MHC in the peptide-loaded complex and affects the resulting affinity of TCR-CD8-MHC complex. In vivo, the CD8-MHC interaction has an influence on T-cell survival and depletion during the thymic selection, while in vitro, it must also be taken into account to avoid over-reactivity of T cells due to promiscuous binding of their TCR and CD8 components to different pMHC complexes.

### 4.2. TILs—Not-Engineered T-Cells

Tumor infiltrating lymphocytes (TILs) represent the oldest branch of ACT, the so-called “blind” approach that includes cultivation, expansion, and subsequent transfusion of TILs for the treatment of tumors. Initially, these cells are isolated from homogenized tumors or sentinel lymph nodes and then cultured with IL-2 in the presence of tumor lysate as a source of tumor-antigens and peripheral blood mononuclear cells (PBMC) for efficient antigen presentation [43]. Finally, following the expansion, TILs suspension could be infused back into the patient as a cell therapy [44]. Similar to CAR T-cells, certain subpopulations of TILs in the infusion product were associated with higher therapy efficacy, e.g., elevated proportion of regular CD8^+^ T-cells, their more differentiated effector phenotype or CD8^+^ T-cells co-expressing the B- and T-lymphocyte attenuator (BTLA) [45].

Mostly, this ACT approach was used against “hot” tumors with high mutational burden (TMB), immunogenicity, and T-cells enrichment (melanoma, colon adenocarcinoma), and typically resulted in 30–50% response rate [43,45,46,47,48], including up to 24% long-lasting complete responses in melanoma clinical trials [48]. Generally, melanoma is highly responsive to various immunotherapies and nearly 40% of metastatic melanoma patients were reported as staying progression-free for over four years after treatment with a combination of ipilimumab (anti-CTLA4) and nivolumab (anti-PD-L1) [49]. In a fraction of patients who relapsed after anti-PD-L1 therapy, TILs retained specific anti-tumor cytotoxicity and their infusion resulted in two partial responses in twelve patients with metastatic melanoma, previously relapsed after check-point inhibitors [50]. This demonstrates the potential of TILs in such a highly resistant cohort of patients and suggests that the results of therapy may be further improved by the optimization of culturing conditions. Indeed, a simple modification, such as the addition of anti-CTLA4 antibody during the expansion increased TIL proliferation, the proportion of CD8^+^ T-cells within the cell product and increased TIL reactivity towards autologous ovarian cancer cell lines in vitro [51]. Moreover, the elevation of potassium levels in the medium during TIL expansion led to the less differentiated T-cells that showed long-term persistence within tumors resulting in prolonged survival and better tumor regression in melanoma mouse model [52]. Other strategies to limit the cell differentiation include pharmacological blockade of phosphoinositide 3-kinase (PI3K) and shortening the duration of ex vivo expansion [29].

However, in other types of tumors, mostly in “cold” tumors lacking infiltrated T-cells, the successful application of TILs might become highly challenging. For example, despite relatively accessible isolation of TILs in pancreatic cancer [53], only a small number of trials with TILs from such tumors is listed on clinicaltrials.gov.

### 4.3. Strategies for Selection of TCR-Dependent Epitopes

Production and therapeutic transfusion of T-cells with known specificity is also utilized beyond the TILs approach. Typically, cancer tissue harbors many somatic mutations, with some of them driving the oncogenesis and others staying as bystander mutations. Nevertheless, the mutations often result in translation of abnormal protein that may be further processed into new immunogenic T-cell epitopes (so called “neoantigen”) and serve as a potential target for the T-cell therapy. The patient’s own peripheral T cells or TILs may be used as a cell source for the antigen-specific expansion or could be transduced with the artificial TCR specific to the neoantigen of choice. Moreover, the abovementioned alternative cell sources such as NK-cells, γδ T-cells from the allogeneic donors may also be used for transduction of the exogenous TCR. The presence of the mutation alone is not sufficient to make a suitable target for the T-cell immunotherapy. Only about 0.0125% of the putative 8–9 amino acids long peptides could be recognized by T-cells in a model of post-infection immunity [54]. The parameters reducing the number of candidate mutations/peptides include: (i) sufficient intracellular expression of the source protein; (ii) the need for appropriate HLA-binding; (iii) efficient proteasomal processing; (iv) immunogenicity (capability of inducing immune response); and (v) recognition of the epitope during infection. Given all the above, identification of suitable neoantigens amenable for T-cell immunotherapy becomes a highly challenging task.

#### 4.3.1. Sequencing Strategies

Whole exome sequencing with or without RNA-sequencing allows the mapping of mutations to the reference genome and, upon combination with mass-spectrometry (MS) of immunopeptidome (HLA-binding peptides), further enables identification of the exact epitopes that were processed by proteasome and presented in HLA molecules [55,56].

Although considered a powerful research tool, sequencing combined with MS still has some shortcomings. For example, it can barely identify low-copy transcripts and therefore fails to determine their impact on the immunopeptidome. Indeed, HLA-eluted peptides that are not mapped to the reference RNA-seq/exome-seq data will be omitted, although some immunogenic epitopes still may be derived from the low-copy transcripts or even non-coding regions. This concept was extensively elaborated theoretically [57] and then confirmed by subsequent experimental studies [58]. Laumont et al. claim that the abundance of neoantigens derived from alternative or non-coding open reading frames (ORFs) is much higher than that of canonical protein-derived antigens [57]. Only about 2% of the human genome encodes proteins, whereas ~75% of it could be found in transcriptome including the transcripts that were identified in MS datasets [59,60].

The immunogenicity of candidate neoantigens and their potential cross-reactivity with healthy tissues should be further assessed. For this purpose, endogenous processing of neoantigens can be evaluated by expansion of T-cells in the presence of autologous APCs pulsed with candidate peptides as individual molecules or tandem minigenes allowing one to check not only allele binding, but also the proteasome cleavage of the candidate peptide [61,62,63].

#### 4.3.2. Bioinformatic Selection

Alternatively to the above-described, the MS complementation of sequencing data may be performed by “in-silico MS”, i.e., prediction of binding the exact HLA allele to the candidate neoantigens derived from the mutated sequence. The prediction is possible using computational tools that use extensive datasets of HLA MS data and paired RNA-sequencing or exome-sequencing. The examples include NetMHCPan (latest version 4.0) [64], SYFPEITHI [65], or HLAthena [56] for MHC class I peptides and NetMHCIIPan (latest version 4.0) [64] for MHC class II peptide binding prediction. Due to the ongoing expansion of the datasets for model learning, the prediction accuracy also constantly increases, reaching up to 75% for HLAthena. Despite the overall high promise, none of these tools alone is suitable for credible prediction of neoepitopes due to the uncertainty in the proteasomal cleavage of the source protein resulting in the generation (or not-generation) of the predicted neoepitope. The accuracy of computational methods in the field of proteasome cleavage prediction is still far from being perfect; however, additional tools such as NetChop or PCPS can significantly improve the data fidelity [66,67,68].

The abovementioned approach was adopted for clinical use by Chen et al., who identified a number of highly frequent mutations shared within various types of human cancers and designed a library of neoantigens predicted to bind common HLA-A alleles [69]. This set of neoantigens covered different proportions of patients across 9 selected cancer types (up to 89.6% of patients with pancreatic cancer, median coverage of 23%, range 9.5–89.6%, HLA-matching was omitted from calculation). Reactivity of peripheral T-cells against at least a single peptide from this dataset was observed in 6 out of 13 patients with various solid tumors. The authors proposed a mixed immunotherapy approach: neoantigen-pulsed dendritic cell vaccine alone or in combination with autologous T-cells (~10^8^ per infusion) expanded for 10–17 days. For 6 treated patients, therapy resulted in 1 complete remission, 1 partial response, and for 4 others, disease stabilization and prolonged survival.

#### 4.3.3. Library-Associated Epitope Screening

**Figure 3:**
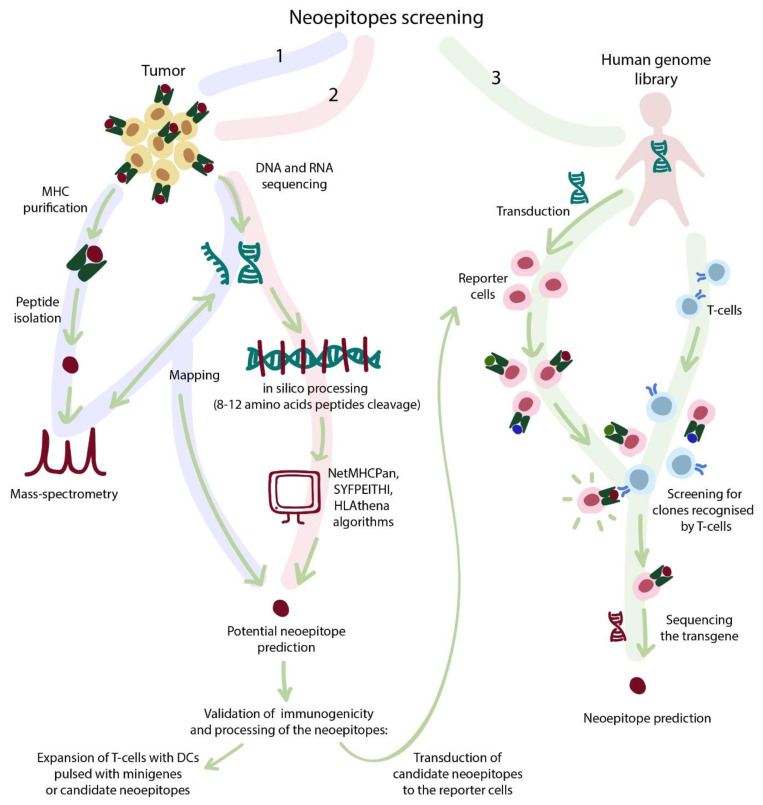
The strategies for neoantigen discovery. (1) A laborious approach that includes: (a) tumor genome and RNA sequencing; followed by (b) MS analysis of the immunopeptidome (peptides dissociated from peptide–MHC complex) and (c) mapping of these peptides to the source genome/transcriptome. (2) Analysis of the sequencing data allows computational prediction of putative HLA-binding peptides by NetMHCPan, SYFPEITHI, or HLAthena algorithms. (3) Novel approaches for epitope screening based on human genome-wide libraries. Approaches 1 and 2 require subsequent validation to confirm correct processing and presentation of the identified peptides (for approach 2), as well as their potential immunogenicity (for both approaches). The validation step includes ex vivo expansion of T-cells in presence of peptide- or minigene-pulsed dendritic cells (DCs) or using approach 3.

The validation of candidate neoantigens is highly laborious and includes one by one checking of processing, presentation, and immunogenicity of each antigen (summarized in Figure 3). Two recently introduced approaches address these issues according to the following procedure: (i) the library of candidate neoantigens is introduced into reporter target cells that process and present those antigens; (ii) T-cells and target cells get cocultured; (iii) a proportion of target cells change the fluorescence pattern, because they are recognized by specific T-cells; (iv) recognized target cells are separated by fluorescence-activated cell sorting (FACS) and sequenced, thus allowing the identification of the neoantigens. Sharma et al. systematically assessed the sensitivity of their approach in terms of the density of specific epitopes and their associated T-cells and found that the epitope frequency of 1:10,000 and target T-cell abundance of 1:30 is optimal, while reduction of T-cell abundance to 1:3000 (with the same epitope frequency) resulted in disappearance of the target epitope in output data [70]. The other research by Kula et al. was based on T-scan technology and focused on the identification of cross-reactive epitopes for TCRs with allegedly known specificity, e.g., to NLV (cytomegalovirus epitope) or MAGE-A3 (melanoma epitope) [71]. They identified three epitopes, cross-reactive for MAGE-A3 TCR with low degree of homology (3–4 amino acids) derived from human genes FAT2, PLD5, and MAGE-A6. This approach showed its utility for identification of cross-reactive epitopes, which is crucially important for development of artificial affinity-matured TCRs described in the next section.

### 4.4. Transgenic TCRs

The introduction of transgenic TCRs potentially allows the adding of antigen-specificity to any T-cell and using them for ACT purposes. The common origins of transgenic TCRs include: (i) sequencing of TIL TCRs with subsequent identification of target epitope by prediction algorithms; (ii) neoantigen-specific in vitro expansion of T-cells isolated from patient or other HLA-matched/mismatched donor; and (iii) extensive TCR mutagenesis aimed to increase affinity or apply other modifications.

**Figure 4:**
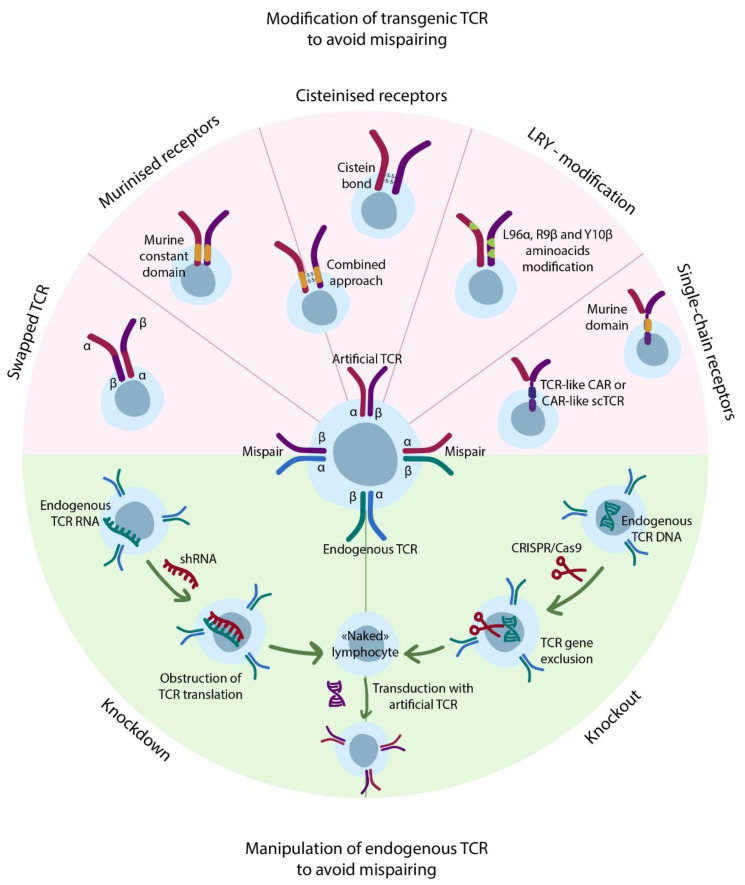
Addressing mispairing issues. The top semicircle (rose) depicts modifications of transgenic TCRs that facilitate correct pairing of ɑ and β chains. The bottom semicircle (green) outlines regulation of the endogenous TCR expression.

In comparison to ex vivo expanded TILs, the transgenic (artificial) TCR T-cell therapy requires genetic modification of the bulk T-cells and usually includes the introduction of TCR α and β chains by means of viral transduction. The significant risk of this approach lies in potential mispairing of the cognate and exogenous TCR chains that might lead to unknown specificity of the mispaired receptors (Figure 4). Nevertheless, to date, no toxicity caused by TCR mispairing was reported in humans, although in mice, lethal GvHD was described [72]. Several approaches were proposed to avoid TCR mispairing. First of all, the knockout [73] of endogenous TCR completely eliminates incorrect pairing issues, but is very labor-intensive and time-consuming and thus would be extremely hard to adapt according to GMP. RNA-mediated knockdown [72,74] of endogenous TCR is easier to combine with common transgene-introducing techniques to achieve significantly reduced endogenous TCR expression and thus mispairing, as was demonstrated in a mouse GvHD model [72]. Despite all efforts, the residual expression of endogenous TCR may persist, thereby carrying a risk of autoimmunity. The other approaches include modification of transgene TCR itself by: (i) replacement of some amino acids with cysteine to form disulfide bond between the chains [75]; (ii) substitution of constant domains of human TCRs with murine ones [76] (both methods were developed by Steven Rosenberg and colleagues); and (iii) combination of single chain TCR (scTCR) cysteinization with expression of additional murine Cα domain [77]. Moreover, Thomas et al. analyzed sequences of dominant (strongly expressed) and weak (weakly expressed) TCRs and identified amino acids essential for enhanced TCR expression in the constant regions of α and β chains [78]. The substitution of other amino acids in certain positions to the ones identified through sequencing (LRY-modification) allowed a 3–6 fold increase in expression of weak TCRs. Moreover, authors found reduced mispairing between LRY-modified transgene and endogenous TCR chains, making this approach potentially useful. In some cases, 3-fold increase in TCR expression levels led to 3000-fold enhancement in T-cell avidity to cognate peptides and even induced recognition of similar peptides differing only in several mismatches. Nevertheless, in clinical settings, such TCR enhancement carries risks of potential cross-reactivity and recognition of low-density tumor-associated antigens in healthy tissues. Another elegant approach addressing mispairing was proposed by Bethune et al., who swapped constant domains of α and β TCR chains therefore inhibiting the formation of functional TCR-CD3 complexes when mispaired with the endogenous TCR chains [79]. In contrast to others, this approach doesn’t reduce the TCR mispairing, although it makes the mispaired receptors non-functional. Such features eliminate potential autoimmunity of TCR-swapped T-cells (as confirmed in mice models) but significantly reduces the density of functional transgenic TCRs. As the authors demonstrate, this method still can be further improved by miRNA-mediated endogenous TCR knockdown.

Completed and ongoing clinical trials exploiting transgenic TCR T-cell therapy with a special focus on the TCR design are listed in Table S1.

The Latest Generations of Artificial TCRs: Addressing Not Only Mispairing. Single Chain TCRs and TCR-Like CARs.

**Figure 5:**
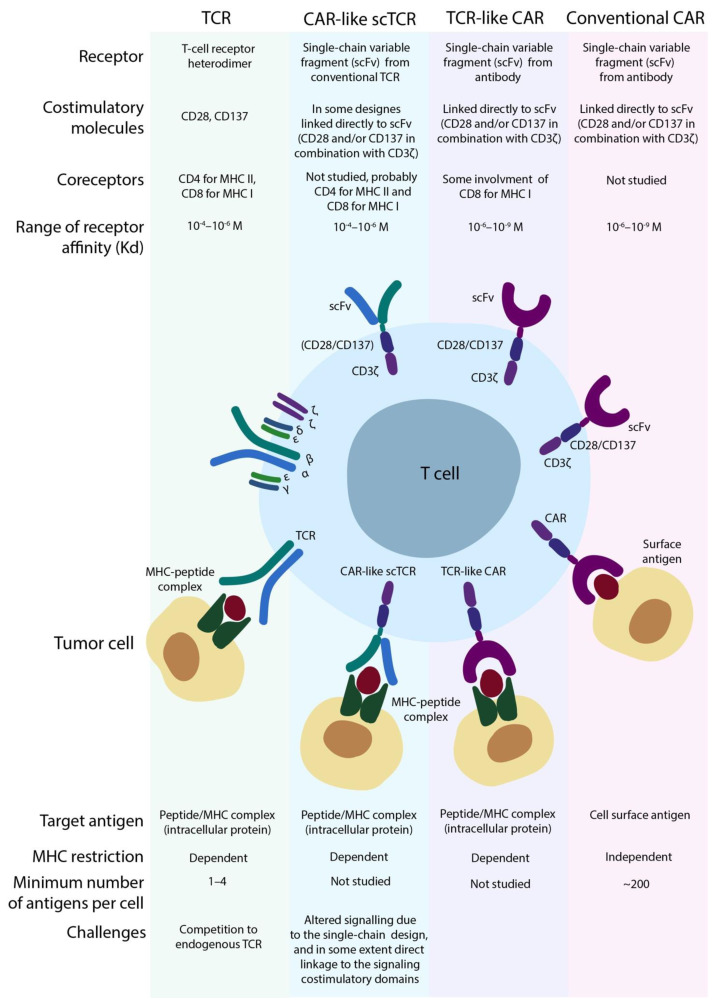
The differences and similarities in the design of transgenic receptors for adoptive immunotherapy.

The boundary between scTCRs and TCR-like CARs is vague and often subjective (Figure 5), and the term “TCR-like CAR” itself is more appropriate for CARs bearing scFv antibody domains specific to pMHC complex. Indeed, initially, scTCRs were designed as three-domain constructs consisting of two variable and one constant domains (β chain) [77,80,81,82,83], sometimes co-expressed with an additional constant α chain for improved signaling [77]. Aggen et al. demonstrated that the above mentioned multi-domain construction doesn’t completely prevent mispairing due to the preserved constant domain [83]. In an attempt to resolve this issue, authors introduced scTCR with two variable domains linked to the construction, very similar to the transmembrane and intracellular domains of the 1st generation CAR (we address this below as CAR-like scTCR). They demonstrated that for this approach, the mispairing was undetectable by flow cytometry. In another study, Zhang et al. extensively studied the different designs of single chain variable domains linked to the diverse transmembrane and intracellular domains and found that surface expression of CAR-like scTCRs on T-cells after retroviral transduction was affected by the origin of the transmembrane (TM) region and position of signaling domains [81]. These scTCR-modified T-cells were functional as confirmed by cytokine (IL-2 and IFN-g) release in response to antigen stimulation and cytolytic activity against specific target cells.

Harris et al. [84] used yeast display assay and designed scTCRs with high affinity to two known tumor-associated antigens (overexpressed in tumor cells, but having low expression in healthy tissues): WT1 (acute myeloid leukemia) and MART (melanoma). They directly compared the designed affinity-maturated conventional TCR with the CAR-like scTCR (named TCR-like CAR), originated from this conventional TCR [84]. They found that T-cells with conventional TCRs were 10–100 fold more sensitive to the antigen compared to TCR-like CARs, despite significantly higher surface expression of the latter. The authors showed that this effect was not associated with reduced affinity of CAR to the peptide-MHC complex. On the opposite, according to the binding curves, the affinity of CAR-pMHC interaction appeared to be nearly two times higher than for conventional TCR. In this case, reduced sensitivity towards the pMHC complex was likely to be associated with altered intracellular signaling. These observations are in good agreement with previous findings that showed a higher activation threshold for CAR T-cells (~200 target molecules per cell) in comparison to T-cells (1–4 targets per cell) [85].

Another interesting scTCR design is based on TCRs that consist solely of the Vβ-chain. Oh et al. demonstrated that, indeed, Vβ-only TCRs (still requiring Cα chain w/o Vα domain for the correct assembly with CD3) had significantly reduced surface expression, possibly due to the mispairing [86]. In contrast to the abovementioned study by Harris et al., these authors found that the sensitivity of scVβ TCRs and CAR-like scTCR towards pMHC complexes was comparable. This was supposedly due to the highly artificial approach for generating scVβ TCRs as well as the use of a different cell model for antigen sensitivity assay. Nevertheless, lower likelihood of mispairing issues makes CAR-like scTCRs somewhat more attractive than conventional scTCRs (with unmodified constant domain) as a prospective antitumor immunotherapy. The specificity and cross-reactivity properties of genetically engineered TCR variable domains may drastically differ from their native progenitors, as summarized in Table 1. Thus, each TCR with modified variable domains has to be tested for potential cross-reactivity. This emphasizes the importance of proper genetic design and comprehensive tests, and may require the introduction of a “safety switch” mechanism for regulation of immune response in the case of developed toxicity. These “safety switch” mechanisms, initially developed for CAR T-cells, include drug-inducible suicide genes, logic gate receptors (AND/OR) [29], as well as ON-switch receptors that require small molecule modulators for multimerization [87]. In addition to the suicide switches, the RNA transfection methods could be applied for initial clinical testing of new TCR and CAR-based therapeutic approaches [88]. Theoretically, this would result in a transient expression of the transgene and reduced probability of off-target complications. The resulting surface levels of the transgene receptor may vary significantly across reported transfection/transduction methods, eventually affecting the avidity of T-cells and thus, their potential autoimmune properties.

### 4.5. Exploiting Alternatives to Conventional TCRs

#### 4.5.1. NK-Cell Receptors

An alternative to NK-cells is the introduction of KIR-based CARs into T-cells. Celyad Inc. is a biotech company that develops both autologous- and allogeneic CAR T-cell products, including NKG2D-based CAR T-cells. Autologous CYAD-01 investigational therapy (THINK clinical trial) for the treatment of AML demonstrated anti-leukemic activity in 6 out of 13 patients [89], and is followed by next generation CYAD-02 that includes short hairpin RNA (shRNA)-mediated knockdown of NKG2D ligands in T-cells (CYCLE-1 Phase I clinical trial). Allogeneic CYAD-101 therapy (alloSHRINK clinical trial) for the treatment of colorectal cancer resulted in 2 out of 12 patients achieving partial remission (PR) and another 5 achieving stable disease (SD) [90]. This cell product expresses TCR Inhibitory Molecule (TIM) peptide that lowers the TCR-associated signaling and thus, the probability of developing GvHD. No dose-limiting adverse events were reported for either of the CAR T-cell therapies.

#### 4.5.2. Non-Conventional TCRs

Recently, Crowther et al. [91] isolated a non-conventional T-cell clone capable of recognizing an unidentified metabolic substance presented in the context of major histocompatibility complex class I-related gene protein (MR1). MR1 is a non-conventional MHC class I molecule highly conservative within the human population, and normally presents metabolites from bacteria and fungi in infected cells [92]. The identified T-cell clone was capable of killing multiple tumor cell lines without any toxicity to the normal tissues, including cells affected by oxidative stress. The TCR of this T-cell clone was sequenced and transduced into human PBMCs and then tested against NSG mouse tumor models. The mice showed prolonged survival and a low number of cancer cells in bone marrow tissue. This particular TCR showed a promising therapeutic potential for HLA-independent treatment of a wide spectrum of cancers [91,93]. Researchers from Cardiff University in partnership with Enara Bio Ltd. are working on a clinical implementation of this type of TCR-directed T-cell therapy against unconventional cancer targets, such as MR1-presented cancer ligands [94].

To summarize, the general TCR-based therapies (e.g., TILs) and the novel promising approaches such as non-conventional MR1-dependent TCRs or NK-cell receptors may be applied for the treatment of a wide range of tumors. New genome-wide screening can assist the identification of multiple novel neoantigens amenable for therapeutic targeting. However, it is important to keep in mind that transgenic TCRs require careful testing for potential cross-reactivity (i.e., resulting in toxicity) and might need additional modifications to prevent mispairing with cognate TCRs. Resolving these issues would make CAR-like scTCRs an attractive alternative to the traditional double-chain TCRs.

## 5. Evolution of Manufacturing Therapeutic T-Cells

**Figure 6:**
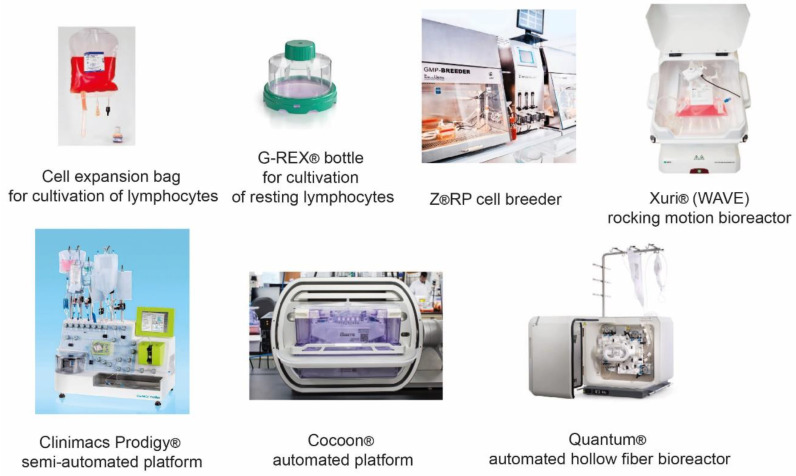
Evolution of the bioreactor platforms. The most advanced systems include G-Rex^®^ by Wilson Wolf, Z^®^RP by Zellwerk, Xuri^®^ (WAVE^®^) by Cytiva, CliniMACS Prodigy^®^ by Miltenyi Biotec, Cocoon^®^ by Lonza (Basel, Switzerland), and Quantum^®^ by Terumo (Tokyo, Japan).

Lymphocyte-based ACTs, such as CAR T-cells, TCR T-cells, NK-cells, and TILs, are demanding in growth conditions and sensitive to mechanical stress; therefore, design of an appropriate suspension culture compatible bioreactor is critical. The evolution of T-cell manufacturing systems began with the static technologies such as T-flasks and simple bag reactors, then proceeded to rocking motion (e.g., WAVE^®^ by Cytiva (Marlborough, MA, USA) bioreactors, and eventually evolved into PoC automated manufacturing systems such as CliniMACS Prodigy^®^ by Miltenyi Biotec (Bergisch Gladbach, Germany) (Figure 6). Single-use bag bioreactors are somewhat similar to the conventional flasks, with the advantage of accurate and sterile medium supply, as well as enhanced sterility during cell growth and various manipulations. Simplicity of the device allows it to be used with the majority of cellular products, e.g., DCs, Tregs, CAR T-cells, TILs [95,96].

The most common bioreactors for growing immune cells are suspension-based, such as the G-Rex^®^ flask by Wilson Wolf Inc. (New Brighton, MN, USA) and Z^®^RP cell cultivation platform by Zellwerk GmbH (Oberkrämer, Germany). The advantages of G-Rex^®^ that led to its wide popularity are the ease of use, simplistic design, and specialized membrane at the bottom of the vessel for intensive gas exchange. G-Rex^®^ devices are particularly relevant for the production of TILs due to their adaptability for scaling up the manufacturing process to perform cell growth in large volumes [97]; although these devices may be successfully applied for manufacturing of CAR T-cells [98] and other types of cellular immunotherapy products.

The Z^®^RP cell breeder for cell and tissue culturing has an unconventional and curious design in which the cell cultivation process takes place in a labyrinthine plastic bioreactor in a static mode. An important feature of this system is a sophisticated perfusion media supply that creates directed and laminar medium flow, which minimizes the impact of mechanical stress on the cells and reduces disturbance of the intercellular contact. In a recent report, Z^®^RP cell cultivation platform was successfully used for growing therapeutic NK-cells in perfusion mode under “steady state” conditions [99].

Most of the rocking motion or wave-induced motion (WIM) bioreactors, including WAVE^®^, are based on a perfusion modality with cells growing in cultivation plastic bags located on a swinging platform. The bag ports are designed to allow continuous medium supply and circulation through a special filter that is not permeable for the cells. The WAVE^®^ bioreactor is often used for cultivation of the majority of therapeutic immune cells [44,100]; however, it was shown to be better suited for manufacturing NK-cells than DCs [101]. Manufacturing of therapeutic macrophages and CAR M-cells can also be performed in suspension with motion-based or stirred tank bioreactors; however, this might require using special low-adhesion materials [102].

A hollow fiber (HF) perfusion bioreactor typically represents a closed cylindrical module containing numerous self-supporting, narrow-bore membrane tubes. In this system, a medium circulates through the bundle of fibers enclosed in a vessel ensuring resupply of nutrients and removal of metabolic waste. Many commercial bioreactors of this type are used to grow adherent cells, such as mesenchymal stem cells (MSCs). However, HF bioreactors, like Terumo’s Quantum^®^ cell expansion system, are also used for suspension cultures, including lymphocytes [103].

One of the most advanced systems for production of immunotherapy cell products (e.g., CAR T-cells) are the semi-automated Myltenyi’s CliniMACS Prodigy^®^ and the fully automated Lonza’s Cocoon^®^ platforms. The CliniMACS Prodigy^®^ system allows isolation of PBMCs from the whole blood, immunomagnetic separation of certain cell subpopulations, followed by their transduction with viral vectors and expansion in the CentriCult Unit that functions both as a centrifuge and as a suspension bioreactor. Currently, the ACT facilities in many academic institutions and clinical centers are based on the CliniMACS Prodigy^®^ system, since it substantially facilitates the manufacturing process by reducing personnel involvement and potential human errors [104].

Lonza’s Cocoon^®^ system is based on a single-use, highly customizable cassette that allows manufacturing CAR T-cell products in a completely autonomous mode with the possibility of scaling up the process for industrial level production. The development of such automated and semi-automated platforms has been actively progressing in recent years to improve the quality and increase the accessibility of ACTs. Further progress in this field will lead to the development of more advanced automated PoC platforms for manufacturing of both allogeneic and autologous cell therapies for personalized immunotherapy.

## 6. Conclusions

CAR T-cells have already revolutionized the treatment of hematological malignancies and now rapidly expand to non-cancer fields such as autoimmune disorders and infectious diseases [105]. ACTs beyond CAR T-cells includes therapies based on CAR NK-cells and γδ T-cells that hold a great promise for large scale manufacturing and universal application. Their eligibility as potential allogeneic therapies require further investigation because γδ T-cell products are considered only as a prospective therapeutic concept, while CAR NK-cells are already being tested in early Phase I clinical trials. The conventional TILs demonstrated encouraging results in highly immunogenic tumors (e.g., melanoma), while for stroma-rich immunologically cold cancers (e.g., pancreatic cancer), more advanced approaches are required, such as CAR M-cells with their outstanding trafficking and epitope spreading. The non-conventional MR1-dependent TCRs and NKG2D CAR T-cells are of high interest for universal tumor targeting. Expanding the range of therapeutic targets becomes possible with novel epitope screening technologies that are highly supportive for the identification and the validation of neoantigens, e.g., T-scan that represents a cell-based, pooled screening assay for high-throughput identification of antigens efficiently recognized by T-cells.

It is generally accepted that a safe application of transgenic TCRs is possible only for infusion of autologous cells originally isolated from the same patient in which the particular TCR was identified. The toxicity due to the off-target recognition of cross-reactive epitopes and the on-target off-tumor recognition of under-expressed epitopes in healthy tissues can result in lethal cases. Therefore, the safety of artificial, affinity maturated and even simply hyper-expressed TCRs must be thoroughly and systematically evaluated prior to wider clinical application. Here, the abovementioned T-scan screening has an outstanding potential of resolving the artificial TCR cross-reactivity issues before the therapeutic application. The other major source of potential toxicity is the mispairing of transgene and cognate TCRs that could be addressed with improved design of the transgene (single-chain TCR, domain-swapped TCR chains, cognate TCR knockdown).

Multiple semi- and fully automated manufacturing platforms are now available on the market and the appropriate system should be carefully selected to meet the specific requirements for each type of cell. Further developments will expand the scope of clinically used therapeutic cell products and increase the affordability and uniformity of ACTs globally.

Overall, the current progress and hopeful results in clinical application of universal, targeted, and combined ACTs for precision medicine and advanced cancer treatment, both within and beyond CAR T-cell therapy, lays a solid foundation for future advances in this field.

## Figures and Tables

**Table 1:** Issues of T-cell receptor (TCR) modification that influence potential cross-reactivity or off-target toxicity.

Modification	Commentary
1. Increased surface TCR levels due to: LRY modification scTCR design Increased CD3 expression	May impact T-cell avidity and recognition of non-target cells with low target antigen levels
2. Limited mispairing between cognate and exogenous TCRs	Prevents cross-reactivity by formation of hybrid TCRs with unknown specificity
3. Affinity maturated TCRs or TCRs with mutated CDR1, CDR2, CDR3 sequences	Unknown additional specificity

## Data Availability

Data sharing not applicable.

## Supplementary Materials

The following are available online at https://www.mdpi.com/2072-6694/13/4/743/s1, Table S1: The list of completed and ongoing clinical trials of transgenic TCR therapies.

## Abbreviations

ACT	Adoptive Cell Transfer
APC	Antigen-Presenting Cell
BTLA	B- and T-lymphocyte attenuator
BTN2A1	Butyrophilin-2A1
CAR	Chimeric Antigen Receptor
CR	Complete Remission
DC	Dendritic Cell
FACS	Fluorescence-Activated Cell Sorting
GMP	Good Manufacturing Practice
GvHD	Graft versus Host Disease
HF	Hollow Fiber
HLA	Human Leukocyte Antigen
HSCT	Hematopoietic Stem Cell Transplantation
IL	Interleukin
Mel	Melanoma
KIR	Killer Cell Immunoglobulin-Like Receptor
MHC	Major Histocompatibility Complex
MRD	Minimal Residual Disease
MS	mass spectrometry
MSC	mesenchymal stem cell
MR1	major histocompatibility complex class I-related gene protein
NK	Natural Killer
NOD/SCID	Nonobese Diabetic/Severe Combined Immunodeficiency
NR	No Response
ORF	Open Reading Frame
PBMC	Peripheral Blood Mononuclear Cell
pMHC	Peptide-loaded Major Histocompatibility Complex
PD	Progressive Disease
PoC	Point-of-Care
PR	Partial Remission
Pt/pts	patient/patients
RNA	Ribonucleic Acid
scTCR	single chain T-Cell Receptor
SD	Stable Disease
shRNA	short hairpin RNA
SCS	Synovial Cell Sarcoma
TCM	Central Memory T-cell
TIL	Tumor Infiltrating Lymphocyte
TM	TransMembrane
TMB	Tumor Mutation Burden
TME	Tumor Microenvironment
Treg	T regulatory cells
TSCM	Stem Memory T-cell
VGPR	Very Good Partial Remission
WIM	Wave-Induced Motion
