# Supplementary material for: Adoptive Immunotherapy beyond CAR T-Cells
Source: Cancers (Basel). 2021 Feb 11;13(4):743. doi: 10.3390/cancers13040743 (PMC7916861; doi:10.3390/cancers13040743)
Supplement: Supplementary file 1 [file cancers-13-00743-s001.pdf]

Table S1. The list of completed and ongoing clinical trials of transgenic TCR therapies.

Target / Disease	Reference	Number of Patients (pt/pts)	Transduced Cells/Vector	Dosage	Cultivation Regimen	Cytokine Supplementation	Clinical Efficacy	Genetic Construct Design/Mispairing Prevention	Toxicity/Suspected Cause
WT1 / myelodysplastic syndrome (MDS), acute myeloblastic leukemia (AML)	[1]	8	PBMC /retroviral vector	2×10^8 - 5×10^9 cells/person	10-14 days	interleukin 2, anti-CD3 antibody, and RetroNectin	3 pts - decrease of blasts<br>5 pts - PD/ SD	TCR from TILs<br><br>endogenous TCR knockdown	In mice and in vitro – cytotoxicity towards podocytes, not confirmed in human during the treatment
WT1 / post-HSCT acute myeloblastic leukemia	[2]	12	EBV+T_{CM} /lentiviral vector	at least one infusion of 10^{10}/m^2 target cells in 7 pts 2nd infusion of 10^{10}/m^2 due to poor persistence	10-14 days after stimulation and transduction, then EBV+ WT1+ cell sorting, next - expansion. Total production time 4-6 week	Protamine sulfate (10 μg/mL), IL-2 (50 IU/mL), IL-21 (30 ng/mL), IL-7 (5 ng/mL), and IL-15 (1 ng/mL)	No relapses among 12 patients compared to 25% relapse probability in historical control	TCR from a healthy donor Cysteinisation of constant regions	No adverse events
WT1 / Myelodysplastic Syndromes (MDS) Acute Myeloid Leukemia (AML)	NCT02550535 [3]	10	PBMC /retroviral vector	Cohort 1 2x10^7 bulk transduced cells /kg<br><br>Cohort 2: 1x10^8 bulk transduced cells /kg	5-7 days	Not specified	All 6 AML pts alive (med follow up 12 months). 3 MDS pts – median survival of 3 months post infusion. 2 died from progressive disease and one from other causes.	Human pWT126-specific TCR	No adverse events apart from one possible cytokine release syndrome
WT1 / AML	NCT02770820	Results not yet disclosed							
MART1 GP100 / melanoma	[4]	MART1 – 20 GP100 - 16	PBMC /retroviral vector	MART1 1.5×10^9 - 1.07×10^{11} cells/patient<br><br>Gp100 1.8×10^9 - 1.1×10^{11} cells /patient	On days 9 to 12, cells were expanded or not an additional 9 to 14 days in 6000 IU IL-2 with 50 ng/mL anti-CD3 mAb OKT-3 and 100-fold excess 5 Gy irradiated allogeneic PBL feeder cells.	IL-2, anti-CD3 mAb OKT-3 and 100-fold excess 5 Gy irradiated allogeneic PBL feeder cells	MART1 30% objective antitumor response<br><br>GP100 19% objective antitumor response	MART1 TCR from TILs No mispairing preventing approaches<br><br>GP100 TCR from immunized mice	In 80% epidermal spongiosis and necrotic epidermal keratinocytes due to the recognition of normal cells expressing the targeted antigen

GP100 / Malignant Melanoma	NCT02889861					Results not yet disclosed			
CEA / metastatic colorectal cancer	[5]	3	PBMC /retroviral vector	$2-4\times10^{8}$ cells/patient	9-12 days	IL-2	1 pt - a 49% reduction in the size of metastases<br>2 pts - NR	TCR from immunized mice	Grade 2/3 diarrhea in 3 pts<br><br>The recognition of normal cells expressing the targeted antigen
CEA / Metastatic cancer	NCT00923806					Results not yet disclosed			
NY-ESO1 / Metastatic Melanoma (Mel), synovial cell sarcoma (SCS)	[6]	Mel – 11 pts<br>SCS – 6 pts	PBMC /retroviral vector	1.6×10^{10} - 1.3×10^{11} cells/patient	9-12 days	IL-2	SCS –<br>4 pts PR<br>2 pts PD<br><br>Mel<br>6 pts PD,<br>3 pts PR,<br>2 pts CR	TCR from TILs<br><br>TCR 1G4-α95:LY: two amino acid ubstitutions in the CDR3α of 1G4 TCR for increased specificity	No adverse events
NY-ESO1 / Multiple myeloma	[7]	20	PBMC /lentiviral vector	2.4×10^{9} cells/patient	9-12 days	anti-CD3/anti-CD28-coated beads Recombinant human IL-2 was added to certain cultures	70% CR<br>10% VGPR<br>10% PR<br>5% SD<br>5% PD	Single and dual CDR3α and CDR2β amino acid substitutions for increased specificity	Some patients had a diarrheal syndrome due to autologous GvHD (not attributable to NY-ESO1-specific T-cells
NY-ESO-1 / Ovarian, fallopian tube, or primary peritoneal cancer	NCT03691376					Results not yet disclosed			
NY-ESO-1 / Advanced NSCLC	NCT03029273					Results not yet disclosed			
NY-ESO-1 / Sarcoma	NCT03462316					Results not yet disclosed			
NY-ESO-1 / synovial sarcoma	NCT04526509					Results not yet disclosed			
NY-ESO-1 and/or LAGE-1a / synovial sarcoma	NCT03967223					Results not yet disclosed			
MAGE-A3 / metastatic melanoma, multiple myeloma	[8]	3	CD25-depleted and monocyte-depleted leukopheresis product lentiviral vector	$2.4 - 5.3\times10^{9}$ cells/patient	9 days	not specified	2 patients died due to cardiac toxicity	The affinity-enhanced MAGE-A3^{a3a} TCR with 4 substitutions in the CDR2α	2 of 3 patient died from cardiac toxicity Cross-reactive epitope from the human protein titin could be potently recognized by MAGE-A3 TCR-engineered T cells.

MAGE A3 / Melanoma and Synovial cell sarcoma	NCT01273181 [9]	10	T cells were separated from PBMC by negative magnetic separation /retroviral vector	3 pts $5x10^9$ - $3x10^{10}$ cells/patient<br>7 pts $3x10^{10}$ - $1x10^{11}$ cells/patient	10 days after first activation and 11-13 days after second activation	stimulation with anti-CD3 monoclonal antibody (mAb), IL-2, and addition of irradiated allogeneic PBMC feeder cells	5 pts – NR<br>3 pts – PR<br>2 pts – CR	TCR from mice was further modified by an Ala118Thr substitution in CDR3α	Severe neurologic toxicity: ransient ischemic attack, coma, seizure, altered mental status, death in 2 patients.<br>High cell dosage in patients with neurotoxity?<br>Cross-reactivity to MAGE-A12 protein?
MAGE-A4 / recurrent esophageal cancer	[10]	10	PBMC /retroviral vector	$2×10^8$ - $5×10^9$ cells/patient	7–10 days	IL2, anti-CD3 antibody, and RetroNectin	8 pts tumor Progression 2 pts stable disease for 26 months objective tumor regression was not observed	TCR from immunized mice	No adverse events
MAGE (various epitopes) / Metastatic renal cancer and melanoma;	NCT03391791					Results not yet disclosed			
HPV-16 E6 HPV+ NHSCC or cervical cancer	NCT03578406 (Recruiting) [11	7	PBMC /retroviral vector	5×106 cells/kg $1×10^7$ cells/kg	Not specified	Not specified	4 pts – SD<br>1 pt - PD	Not specified Arm2: anti-PD1 scFv expressing HPV16 TCR-T cells	Leukocytopenia (100%), anemia (100%), fatigue (60%), fever (40%) and thrombocytopenia (40%).<br>No CRS was observed.
HPV-16 E6 / HPV-associated cancer (Vaginal, anal, head and neck SCC)	NCT02280811 [11]	12	PBMC /retroviral vector	$1x10^9$ – $1,7x10^{11}$ cells/kg	6-9 days	IL-2	7 pts PD<br>3 pts SD<br>2 pts PR	E6 TCR	No autoimmune adverse events or off-target toxicities attributable to E6 TCR T cells
p53 / Metastatic cancer that overexpresses p53 Metastatic Melanoma & Other Metastatic Cancers	NCT00393029 [12]	10	PBMC retroviral vector	$5x10^8$ – $2.8x10^{10}$ cells/patient	6-9 days	Complete medium and AIM-V (1:1 ratio) with OKT3 (30ng/ml) and IL-2 (300 IU/ml)	9 pts NR<br>1 pts PR	Human TCR α and β chains specific for p53	No adverse events
HA-1 / Relapsed or refractory acute leukemia after donor stem cell	NCT03326921					Results not yet disclosed			
HA-1H / Relapsed/Persistent Hematologic Malignancies	NCT04464889					Results not yet disclosed			

CMV antigen / Hematological malignancies and CMV infection	NCT02988258	Results not yet disclosed
CMV antigen / Hematological malignancies and CMV infection	NCT02988258	Results not yet disclosed
HPV E7 / Human papillomavirus-associated cancers	NCT02858310	Results not yet disclosed
HPV16 E7 / Relapsed/Refractory HPV16+ Cancers	NCT03912831	Results not yet disclosed
HPV E7 / Vulvar High-Grade Squamous Intraepithelial Lesions	NCT03937791	Results not yet disclosed
HPV E7 / Cervical Cancer	NCT04476251	Results not yet disclosed
HPV E7 / High-Grade Cervical Intraepithelial Neoplasia	NCT04411134	Results not yet disclosed
HPV E7/ HPV-Associated Oropharyngeal Cancer	NCT04015336	Results not yet disclosed
HBV antigen (not specified) / Hepatocellular carcinoma	NCT02719782	Results not yet disclosed
TGFbII / Metastatic colorectal cancer	NCT03431311	Results not yet disclosed
MCPyV / Metastatic or unresectable Merkel cell cancer	NCT03747484	Results not yet disclosed
TRAIL / Metastatic renal cancer	NCT00923390	Results not yet disclosed
PRAME / AML, MDS or metastatic uveal melanoma	NCT02743611	Results not yet disclosed
EBV antigen / Recurrent or metastatic NPC	NCT03648697	Results not yet disclosed
KRAS / KRAS G12V + tumor	NCT03190941	Results not yet disclosed
KRAS / G12D + tumor	NCT03745326	Results not yet disclosed

Mutant KRAS G12V / Advanced Pancreatic Cancer	NCT04146298	Results not yet disclosed
AFP / Unresectable Hepatocellular Carcinoma	NCT03971747	Results not yet disclosed
AFP / Unresectable Hepatocellular Carcinoma	NCT04368182	Results not yet disclosed
PD1 / EBV-Positive NHSCC	NCT04139057	Results not yet disclosed

References

1. Tawara, I.; Kageyama, S.; Miyahara, Y.; Fujiwara, H.; Nishida, T.; Akatsuka, Y.; Ikeda, H.; Tanimoto, K.; Terakura, S.; Murata, M.; et al. Safety and persistence of WT1-specific T-cell receptor gene2transduced lymphocytes in patients with AML and MDS. *Blood* **2017**, *130*, 1985–1994, doi:10.1182/blood-2017-06-791202.
2. Chapuis, A.G.; Egan, D.N.; Bar, M.; Schmitt, T.M.; McAfee, M.S.; Paulson, K.G.; Voillet, V.; Gottardo, R.; Ragnarsson, G.B.; Bleakley, M.; et al. T cell receptor gene therapy targeting WT1 prevents acute myeloid leukemia relapse post-transplant. *Nat. Med.* **2019**, *25*, 1064–1072, doi:10.1038/s41591-019-0472-9.
3. Morris, E.C.; Tendeiro-Rego, R.; Richardson, R.; Fox, T.A.; Sillito, F.; Holler, A.; Thomas, S.; Xue, S.-A.; Martínez-Dávila, I.A.; Nicholson, E.; et al. A Phase I Study Evaluating the Safety and Persistence of Allorestricted WT1-TCR Gene Modified Autologous T Cells in Patients with High-Risk Myeloid Malignancies Unsuitable for Allogeneic Stem Cell Transplantation. *Blood* **2019**, *134*, 1367–1367, doi:10.1182/blood-2019-128044.
4. Johnson, L.A.; Morgan, R.A.; Dudley, M.E.; Cassard, L.; Yang, J.C.; Hughes, M.S.; Kammula, U.S.; Royal, R.E.; Sherry, R.M.; Wunderlich, J.R.; et al. Gene therapy with human and mouse T-cell receptors mediates cancer regression and targets normal tissues expressing cognate antigen. *Blood* **2009**, *114*, 535–546, doi:10.1182/blood-2009-03-211714.
5. Parkhurst, M.R.; Yang, J.C.; Langan, R.C.; Dudley, M.E.; Nathan, D.A.N.; Feldman, S.A.; Davis, J.L.; Morgan, R.A.; Merino, M.J.; Sherry, R.M.; et al. T cells targeting carcinoembryonic antigen can mediate regression of metastatic colorectal cancer but induce severe transient colitis. *Mol. Ther.* **2011**, *19*, 620–626, doi:10.1038/mt.2010.272.
6. Robbins, P.F.; Morgan, R.A.; Feldman, S.A.; Yang, J.C.; Sherry, R.M.; Dudley, M.E.; Wunderlich, J.R.; Nahvi, A.V.; Helman, L.J.; Mackall, C.L.; et al. Tumor regression in patients with metastatic synovial cell sarcoma and melanoma using genetically engineered lymphocytes reactive with NY-ESO-1. *J. Clin. Oncol.* **2011**, *29*, 917–924, doi:10.1200/JCO.2010.32.2537.
7. Rapoport, A.P.; Stadtmauer, E.A.; Binder-scholl, G.K.; Vogl, D.T.; Lacey, S.F.; Badros, A.Z.; Garfall, A.; Finklestein, J.; Kulikovskaya, I.; Sinha, S.K.; et al. NY-ESO-1-specific TCR-engineered T cells mediate sustained antigen-specific antitumor effects in myeloma. **2016**, *21*, 914–921, doi:10.1038/nm.3910.NY-ESO-1.
8. Linette, G.P.; Stadtmauer, E.A.; Maus, M.V.; Rapoport, A.P.; Levine, B.L.; Emery, L.; Litzky, L.; Bagg, A.; Carreno, B.M.; Cimino, P.J.; et al. Cardiovascular toxicity and titin cross-reactivity of affinity-enhanced T cells in myeloma and melanoma. *Blood* **2013**, *122*, 863–871, doi:10.1182/blood-2013-03-490565.
9. Morgan, R.A.; Chinnasamy, N.; Abate-daga, D.D.; Gros, A.; Robbins, F.; Zheng, Z.; Feldman, S.A.; Yang, J.C.; Sherry, R.M.; Phan, Q.; et al. Cancer regression and neurologic toxicity following anti-MAGE- A3 TCR gene therapy Richard. *J Immunother.* **2014**, *36*, 133–151, doi:10.1097/CJI.0b013e3182829903.
10. Kageyama, S.; Ikeda, H.; Miyahara, Y.; Imai, N.; Ishihara, M.; Saito, K.; Sugino, S.; Ueda, S.; Ishikawa, T.; Kokura, S.; et al. Adoptive transfer of MAGE-A4 T-cell receptor gene-transduced lymphocytes in patients with recurrent esophageal cancer. *Clin. Cancer Res.* **2015**, *21*, 2268–2277, doi:10.1158/1078-0432.CCR-14-1559.
11. Doran, S.L.; Stevanović, S.; Adhikary, S.; Gartner, J.J.; Jia, L.; Kwong, M.L.M.; Faquin, W.C.; Hewitt, S.M.; Sherry, R.M.; Yang, J.C.; et al. T-cell receptor gene therapy for human papillomavirus-associated epithelial cancers: A first-in-human, phase I/II study. *J. Clin. Oncol.* **2019**, *37*, 2759–2768, doi:10.1186/s40425-019-0678-x.
12. Davis, J.L.; Theoret, M.R.; Zheng, Z.; Lamers, C.; Rosenberg, S.A.; Morgan, R.A. Development of Human Anti-Murine T-cell Receptor Antibodies in Both Responding and Non-responding Patients Enrolled in TCR Gene Therapy Trials. *Clin Cancer Res.* **2010**, *16*, 5852–5861, doi:10.1158/1078-0432.CCR-10-1280.
